# External validation of a therapeutic window for risperidone in children with autism spectrum disorder

**DOI:** 10.1002/bcp.70130

**Published:** 2025-08-03

**Authors:** Rebecca A. Hermans, Kathalijne Bruens, Karin M. Egberts, Birgit C. P. Koch, Bram Dierckx, Manon H. J. Hillegers, Marcel Romanos, Regina Taurines, Stefanie Fekete, Khatia Eisenecker, Hans‐Willi Clement, Christian Fleischhaker, Brenda C. M. de Winter

**Affiliations:** ^1^ Department of Hospital Pharmacy Erasmus University Medical Center Rotterdam the Netherlands; ^2^ Department of Child and Adolescent Psychiatry/Psychology Erasmus University Medical Center Rotterdam the Netherlands; ^3^ Rotterdam Clinical Pharmacometrics Group Erasmus University Medical Center Rotterdam the Netherlands; ^4^ Department of Child and Adolescent Psychiatry, Psychosomatics and Psychotherapy University Hospital Wurzburg Germany; ^5^ Department of Child and Adolescent Psychiatry and Psychotherapy University Medical Center Freiburg Germany

**Keywords:** paediatrics, pharmacodynamics, pharmacokinetics, risperidone, therapeutic drug monitoring

## Abstract

Although risperidone is an effective pharmacological intervention for managing disruptive behaviour in children with autism spectrum disorder, it may induce metabolic side effects. This study aimed to externally validate the population pharmacokinetic (popPK) model and the therapeutic window of 3.5–7 ng/mL of risperidone and 9‐OH‐risperidone, developed with data of the SPACe Study. For this external validation, data from the German Therapeutic Drug Monitoring (TDM) Service and TDM‐VIGIL Study was used in nonlinear mixed‐effects modelling to evaluate popPK model performance and in receiver operating curve analyses to define the therapeutic window. Population predictions of the popPK model showed underprediction for risperidone concentrations, but individual predictions were fairly accurate. Receiver operating curve analyses resulted in a therapeutic window of 5.0–8.0 ng/mL. The popPK model seems suitable for use in TDM. Because the current analysis included only a few low sum trough concentrations, we suggest maintaining the therapeutic window of 3.5–7.0 ng/mL.

**What is already known about this subject**

- Risperidone is effective in reducing behavioural problems associated with autism spectrum disorder, but is associated with clinically significant weight gain and other side effects.
- The SPACe Study found that risperidone sum trough concentrations predict weight gain and effectiveness, and a preliminary therapeutic window of 3.5–7.0 ng/mL was established. This window was significantly lower than previously calculated reference ranges.

**What this study adds**

- We externally validated the population pharmacokinetic model for risperidone and 9‐OH‐risperidone created with data from the SPACe Study, which means that this model can be used for a wider paediatric population.
- We also performed an external validation of the therapeutic window from the SPACe Study, which strengthened the evidence for a low range in risperidone sum trough concentrations in children with autism spectrum disorder. The therapeutic window will be further validated in a randomized controlled trial.

## 1 INTRODUCTION

Autism spectrum disorder (ASD) is a developmental disorder characterized by deficits in communication and repetitive behavioural patterns, causing significant impairment in social functioning.^1^ To manage comorbid irritability and aggression, atypical antipsychotic drugs are often prescribed, with risperidone as first choice. The effectiveness of risperidone has been extensively studied.^2^ The Dutch and German guidelines for children advise a starting dose of 0.01 mg/kg/day in children with ASD, which may increase to a maximum of 3.0 mg/day.^3^, ^4^ Although risperidone is effective, it also stimulates the metabolic system, often inducing weight gain and other side effects, such as elevated prolactin and lipid levels and increased fat mass.^5^

Risperidone shows linear pharmacokinetics (PK). It is rapidly absorbed after oral administration, extensively metabolized in the liver by CYP2D6 into its active metabolite 9‐OH‐risperidone, and primarily renally excreted. In extensive metabolisers, the apparent half‐life of risperidone is 3 h and that of 9‐OH‐risperidone is 21 h.

Based on current evidence, therapeutic drug monitoring (TDM) of risperidone is recommended for dose optimisation in adults with psychotic disorders and a therapeutic window of 20–60 ng/mL has been reported.^6^ In children and adolescents, therapeutic reference ranges of 9–33 and 8–26 ng/mL have been suggested for treatment of psychotic disorders and impulsive‐aggressive symptoms, respectively.^7^, ^8^ The concentrations in these windows are the sum of the trough concentrations of risperidone and 9‐OH‐risperidone, commonly referred to as the active moiety.

In the SPACe Study, we built a population pharmacokinetic (popPK) model for risperidone and 9‐OH‐risperidone, and linked sum trough concentrations to both weight gain and effectiveness.^9^ PK was described with a 2‐compartment model for risperidone and a 1‐compartment model for 9‐OH‐risperidone, with bodyweight included as covariate through allometric scaling. We calculated a therapeutic window for sum trough concentrations of 3.5–7.0 ng/mL.^10^ To be able to draw conclusions on the generalisability of the results from the SPACe Study, the current study aimed to externally validate both the popPK model and subsequently the therapeutic window, resulting from this model.

## 2 METHODS

### 2.1 Data collection

Data were obtained from the TDM‐VIGIL study, which included a subproject in the form of a *large simple trial* (EudraCT 2013–004881‐33), which aimed to investigate the (long‐term) safety of antidepressants and antipsychotics in children and adolescents including standardized patient assessment and TDM,^11^ and the laboratory of TDM of the Center of Mental Health of the University Hospital of Wurzburg, which has a routine TDM Service where risperidone concentrations are analysed upon request of the psychiatrist (collected between August 2014 and February 2022). Variables collected were: sex; age in months; ICD10 diagnoses; height; weight; risperidone dosing regimen and time of last intake before sampling; information on steady state; risperidone and 9‐OH‐risperidone blood levels; score on the Clinical Global Impressions (CGI) scale; and comedication. For the TDM‐VIGIL participants, reason for dosing change and a score on compliance were also given. For TDM Service patients, time of sampling was also known, while this had to be estimated for the TDM‐VIGIL patients, based on the information that sampling was done before breakfast and before first medication intake.

### 2.2 Study population

Patients were treated in child and adolescent psychiatry (outpatient) clinics in Germany and Switzerland. Children and adolescents aged 6–18 years were included for analysis. Patients were excluded if steady‐state conditions for blood sampling were not fulfilled or if relevant data were missing (e.g. dosing interval, plasma concentrations). All remaining patients were included for the popPK model validation. For the therapeutic window analysis, patients were excluded if they used risperidone more than twice daily. For analysis of the upper cut‐off, further exclusion criteria were a diagnosis of bulimia nervosa or anorexia nervosa, because of their influence on weight, and missing starting dose, starting date, or baseline height and weight. For analysis of the lower cut‐off, patients were included if they had an ICD10 diagnosis of F84.0 or F90.1,^12^ corresponding to pervasive developmental disorders and attention‐deficit hyperactivity disorders, or a diagnosis for ASD according to the Diagnostic and Statistical Manual of Mental Disorders, Fifth Edition.^13^ Patients who did not have 1 of these diagnoses were excluded, as we suspect that different therapeutic windows exist for different treatment indications, such as psychosis.

### 2.3 Drug concentration measurements

Risperidone dosing regimen and comedication were reported during sampling. For TDM‐VIGIL samples, predose samples were taken and the time of risperidone intake in the prior 24 h was reported. For TDM Service, samples were taken at random time points and time of last risperidone intake was according to dose administration times in the Wurzburg hospital. All serum samples were analysed at the laboratory of TDM in the Center of Mental Health of the University Hospital of Wurzburg with high performance liquid chromatography. The lower limit of detection for both risperidone and 9‐OH‐risperidone was 3.0 ng/mL.

### 2.4 PopPK model validation

For the popPK model validation, nonlinear mixed‐effects modelling was performed using NONMEM version 7.4.4 and PsN Version 5.0.0 in Pirana software version 3.0.0, an interface between NONMEM and R (version 4.3.2). Concentrations of 9‐OH‐risperidone were corrected for the molecular weight of risperidone using the formula: (concentration/426.5)*410.5.^14^, ^15^ The PK parameter estimates of the risperidone model form the SPACe Study were used as fixed estimates.^9^ To evaluate model performance, goodness‐of‐fit (GoF) plots and visual predictive checks (VPCs) were performed (*n* = 1000).^16^

### 2.5 Therapeutic window validation

#### 2.5.1 Upper cut‐off

The upper cut‐off of the therapeutic window was based on difference in body mass index (BMI) between baseline and follow‐up. Standardized BMI z‐scores were calculated using the reference table from the World Health Organization.^17^ For the original therapeutic window, a BMI z‐score increase >0.5 between baseline and 6‐months follow‐up was used as a cut‐off value for significant weight gain.^10^ In the current dataset, the following thresholds for BMI z‐score increase were used: ≤1 month: +0.1; 1–2 months: +0.2; 2–3 months: +0.3; 3–5 months: +0.4; and ≥5 months: +0.5., as BMI increase is greater in the first few weeks of treatment,^18^, ^19^ If there were multiple follow‐up measurements, we aimed for a follow‐up as close to 6 months as possible with a dose that was representative for that patient.

#### 2.5.2 Lower cut‐off

The lower cut‐off was based on treatment effectiveness assessed with the CGI scale, filled out by the treating physician. Patients' conditions were rated as 0: not assessable; 1: very much improved; 2: much improved; 3: minimally improved; 4: unchanged; 5: minimally worse; 6: much worse; or 7: very much worse. CGI scores of 1 and 2 were seen as clinically significant reduction of symptoms.^20^ If there were multiple measurements, the same time point was chosen as for the assessment of the BMI z‐score.

### 2.6 Statistical analyses

Statistical analyses were performed with R. Continuous variables were described as the median (interquartile range, IQR). Statistical significance was defined as *P* < .05. Two sampled *t*‐tests were performed to assess the difference in risperidone concentrations between groups with and without significant weight gain and clinical improvement. Cut‐offs of the therapeutic window were determined using receiver operating characteristic (ROC) curves.

## 3 RESULTS

### 3.1 Study sample

In total, 48 patients of the TDM‐VIGIL study and 151 from the TDM Service database were included. Data of 46 patients were suitable for further analyses, among whom only 10 children had sufficient available information to be included in the lower and upper cut‐off analyses. Baseline characteristics of the 3 patient samples are shown in Table 1.

**TABLE 1:** Baseline characteristics per analysis.

Characteristic	PK model validation	Analysis of lower cut‐off	Analysis of upper cut‐off
	*N* = 46	*N* = 10	*N* = 10
**Sex** ^a^			
Female	11 (23.9%)	1 (10.0%)	0 (0%)
Male	35 (76.1%)	9 (90.0%)	10 (100.0%)
**Age** ^b^ **(years)**	14.50 (11.10, 16.80)	11.90 (9.43, 14.80)	10.20 (8.95, 13.63)
**Height** ^b^ **(m)**	1.61 (1.48, 1.73)	1.46 (1.30, 1.62)	1.43 (1.29, 1.58)
**Weight** ^b^ **(kg)**	53 (40, 67)	40 (31, 55)	35 (29, 52)
**BMI** ^b^ **(kg/m** ^**2**^ **)**	20.2 (17.7, 23.0)	17.9 (15.9, 19.9)	17.9 (16.1, 20.3)
**BMI‐z score** ^b^	0.47 (−1.00, 1.35)	0.65 (0.04, 0.87)	0.33 (−0.38, 0.73)
**Comedication** ^a^			
Methylphenidate	13 (28.3%)	4 (40.0%)	3 (33.3%)
Amphetamine	1 (2.17%)	1 (10.0%)	1 (10.0%)
Atomoxetine	2 (4.35%)	0 (0%)	0 (0%)
**Diagnoses (ICD10), including comorbidities** ^a^			
Behavioural and emotional disorders (F90–F98)	23 (50.0%)	8 (80.0%)	8 (80.0%)
Intellectual disabilities (F70–F79)	7 (15.2%)	0 (0%)	1 (10.0%)
Mental disorders due to physiological conditions (F01–F09)	1 (2.17%)	0 (0%)	0 (0%)
Mood (affective) disorders (F30–F39)	8 (17.4%)	3 (30.0%)	2 (20.0%)
Nonmood psychotic disorders (F20–F29)	8 (17.4%)	0 (0%)	1 (10.0%)
Nonpsychotic mental disorders (F40–F48)	4 (8.70%)	1 (10.0%)	2 (20.0%)
Pervasive and specific developmental disorders (F80–F89)	11 (23.9%)	2 (20.0%)	0 (0%)
Unspecified mental disorder (F99)	2 (4.35%)	0 (0%)	0 (0%)

^a^ Frequency (%).

^b^ Median (interquartile range).

### 3.2 PopPK model validation

**FIGURE 1:**
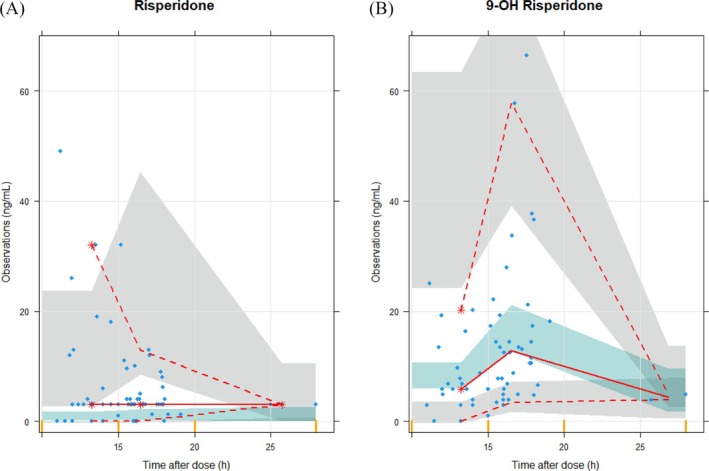
Visual predictive check of validation of the pharmacokinetics model stratified into a compartment for risperidone and 9‐OH‐risperidone. Visual predictive check showing the 50th percentile of the observations as the solid red line and 2.5th and 97.5th percentiles as the 2 dashed red lines and the 95% CI of the model simulated 50th (blue shaded area) and 2.5th and 97.5th percentiles (grey shaded areas).

Model validation was performed with data from 46 patients, with 68 risperidone concentration measurements with a median (IQR) of 3.0 ng/mL (3.0–5.0) and 64 9‐OH‐risperidone concentrations with a median (IQR) of 8.0 ng/mL (4.9–15.0). Median (IQR) dose was 1.50 mg (1.00–2.00). The model performed better for 9‐OH‐risperidone concentrations compared to parent substrate risperidone in each visualization in the GoF plots (Figure S1) as well as in the VPC (Figure 1). Risperidone was often underpredicted, while 9‐OH‐risperidone was sometimes overpredicted.

### 3.3 Therapeutic window validation

#### 3.3.1 Upper cut‐off

**FIGURE 2:**
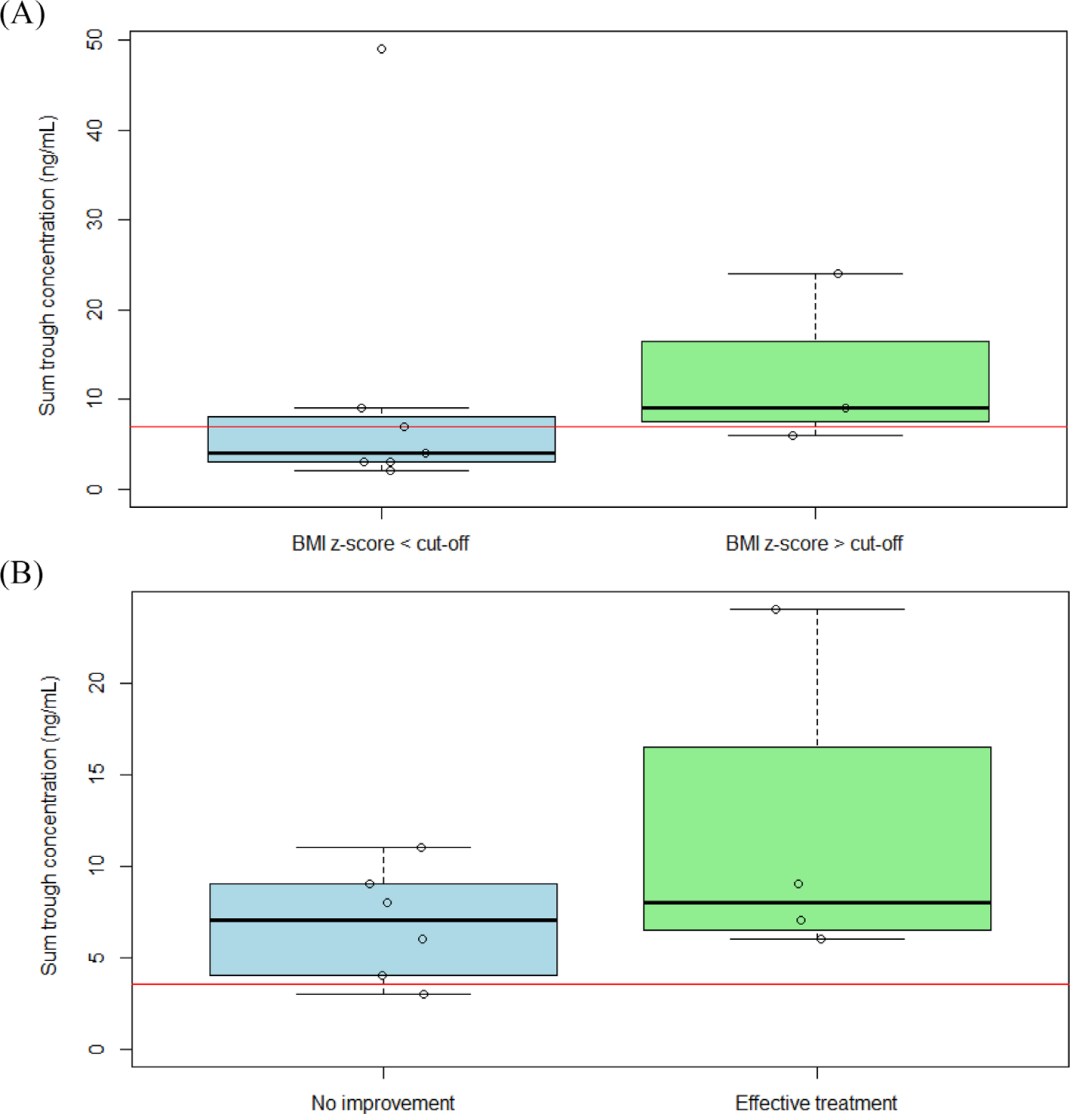
Boxplots showing risperidone sum trough concentrations in (A) patients with BMI z‐scores below and above the cut‐off based on follow‐up time, and (B) patients with and without treatment effectiveness. The red horizontal lines represent the cut‐off values of the therapeutic window from the SPACe study.

The 10 participants in the sample for upper cut‐off analysis used a median (IQR) dose of 0.75 mg (0.50–1.00) and had a median (IQR) risperidone sum trough concentration of 6.5 ng/mL (3.3–9.0). Visually, using the cut‐off values based on the follow‐up period for BMI z‐score increase, higher sum trough concentrations were observed in the 7 patients with significant weight gain (Figure 2A), but this was not significant (*P* = .86). The ROC analysis resulted in a 5.0 ng/mL cut‐off (area under the curve = 0.74).

#### 3.3.2 Lower cut‐off

The 10 participants in the sample for lower cut‐off analysis used a median (IQR) dose of 0.63 mg (0.50–1.38) and had a median (IQR) risperidone sum trough concentration of 7.5 ng/mL (6.0–9.0). The boxplot in Figure 2B shows that the 6 patients with effective treatment often had a higher sum concentration, but this was not significant (*P* = .24). All concentrations apart from 1 were above the cut‐off from the SPACe Study (3.5 ng/mL). The ROC analysis resulted in a 5.0 ng/mL cut‐off (area under the curve = 0.67).

#### 3.3.3 Therapeutic window

Since both cut‐offs were defined at the same sum trough concentration, the data were further evaluated. For the upper cut‐off, there was 1 patient with a sum trough concentration of 6 ng/mL and significant BMI z‐score increase, while the next 2 higher sum trough concentrations did not correspond to significant weight gain. The point on the ROC curve second furthest away from the sensitivity/specificity line corresponded to a sum trough concentration of 8.0 ng/mL. Thus, the therapeutic window was defined as 5.0–8.0 ng/mL.

## 4 DISCUSSION

This study aimed to externally validate the popPK model and therapeutic window for risperidone as defined in the SPACe Study.

Using GoF and VPC to validate the popPK model, we found a better prediction performance for 9‐OH‐risperidone compared to risperidone. Overall, we found that population predictions from the model underpredicted risperidone concentrations and overpredicted 9‐OH‐risperidone concentrations.

These results are consistent with a study by Karatza *et al*., which described an external validation of the SPACe popPK model, along with 1 adult and 2 other paediatric risperidone PK models.^21^ This validation observed an underprediction trend for risperidone in all 4 models, but described the SPACe model as an effective predictor for 9‐OH‐risperidone concentrations. The popPK model's individual predictions were fairly accurate, which means that it seems suitable for use in TDM.

Regarding the therapeutic window, we ultimately defined a range of 5.0–8.0 ng/mL. As the upper and lower cut‐offs using Youden's index were the same at 5.0 ng/mL, which would not be clinically feasible to adhere to, the second‐best point on the ROC curve was chosen for the upper cut‐off. This indicates that it is preferable to stay within the lower values of the range to limit BMI z‐score increase.

The therapeutic window defined with this dataset is comparable to the earlier calculated range of 3.5–7.0 ng/mL. The current analyses were performed with 2 very small samples and with relatively few low sum trough concentrations. For example, only 1 sum trough concentration between 3.5 and 5.0 ng/mL was included in the analysis for the lower cut‐off. For this reason, we would not change the therapeutic window of 3.5–7.0 ng/mL based on these analyses, but instead view the comparable result as a validation of the therapeutic window for children with ASD being significantly lower than the previously calculated reference ranges for adults as well as children.^6^, ^7^, ^8^

This study has several limitations. Most importantly for the popPK model validation, we received data that was not collected with this analysis in mind, meaning that some assumptions had to be made. This concerned time of blood sampling and steady state conditions for some patients. While differences to reality are most likely small and measurements were excluded from analysis if the data contradicted these assumptions or there were too many unknown factors, this could have had an impact on the model validation. For example, the few high risperidone trough concentrations might have actually been measured after medication intake. ROC curve analyses were mostly hindered by the small sample size of 10 patients per analysis. This meant that every single measurement had a great impact on the selection of the cut‐off values, which should thus be interpreted with caution. We took this into account by not only using Youden's index but also looking at all data points to establish the cut‐offs. Another limitation is that the TDM‐VIGIL study adjusted risperidone dosages for some patients based on the therapeutic window of 20–60 ng/mL in adults.^6^ While dosing advice was solely based on this window and clinical improvement was more important than reaching the correct range, meaning that patients who already experienced sufficient effectiveness did not increase their dosage, it could have led to higher doses than necessary to achieve treatment effectiveness.

This study provides a stronger basis for the use of the popPK model and therapeutic window developed in the SPACe study in further research. Currently, in the SPACe 2: STAR study, we are using the popPK model to estimate trough levels based on concentrations sampled at random time points and providing dosing advice based on the therapeutic window of 3.5–7.0 ng/mL.^22^ Data collected during this study should be used to further improve the popPK model and will serve as further validation of the therapeutic window. For now, the tendency of the popPK model to underpredict risperidone concentrations should be kept in mind when giving TDM based dosing advice.

## AUTHOR CONTRIBUTIONS

BK, BD, and BW designed and were investigators on the SPACe Study. MR, KEg, RT, SF, HC, and CF designed and were investigators on TDM‐VIGIL. BK, BD, BW, RH, KEg, and MH designed this collaborative research project. KB and RH performed analyses, and KEg and KEi performed verification of the German data. KB wrote the first draft of the manuscript. RH wrote the second draft and subsequent versions. All authors contributed to subsequent drafts and gave final approval of the version to be published.

## CONFLICT OF INTEREST STATEMENT

The authors have no conflicts of interest.

## CLINICAL TRIAL REGISTRATION

TDM‐VIGIL is registered in the EudraCT database, number 2013–004881‐33.

## Supporting information


**FIGURE S1.** Goodness‐of‐fit plots of validation of the pharmacokinetics model stratified into a compartment for risperidone and 9‐OH‐risperidone.

## Data Availability

The data supporting this study's findings are available from the corresponding author upon reasonable request.
