# Supplementary material for: External validation of a therapeutic window for risperidone in children with autism spectrum disorder
Source: Br J Clin Pharmacol. 2025 Aug 3;91(10):3009–15. doi: 10.1002/bcp.70130 (PMC12464617; doi:10.1002/bcp.70130)
Supplement: Supplementary file 1 — FIGURE S1. Goodness‐of‐fit plots of validation of the pharmacokinetics model stratified into a compartment for risperidone and 9‐OH‐risperidone. [file BCP-91-3009-s001.docx]

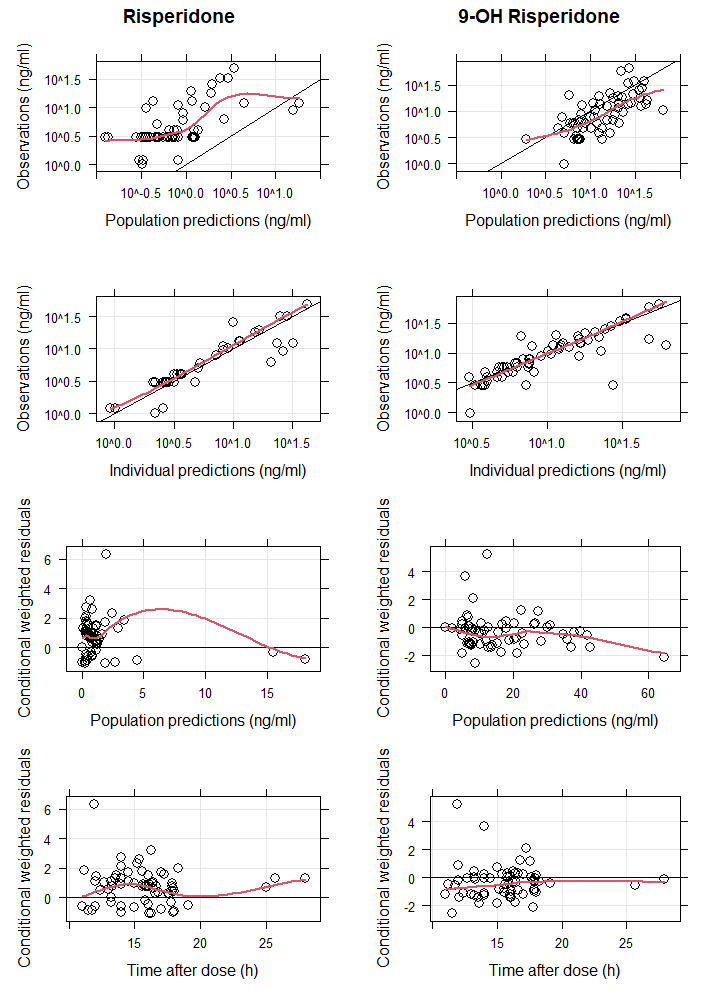


**Supplementary Figure 1. Goodness of Fit plots of validation of the pharmacokinetics model stratified into a compartment for risperidone and 9-OH-risperidone.**
